# Interleukin 10 Polymorphisms as Risk Factors for Progression to Chagas Disease Cardiomyopathy: A Case-Control Study and Meta-Analysis

**DOI:** 10.3389/fimmu.2022.946350

**Published:** 2022-07-04

**Authors:** Alicia Grijalva, Lucia Gallo Vaulet, Roberto Nicolas Agüero, Analia Toledano, Marikena Guadalupe Risso, Juan Quarroz Braghini, David Sosa, Paula Ruybal, Silvia Repetto, Catalina Dirney Alba Soto

**Affiliations:** ^1^ Instituto de investigaciones en Microbiología y Parasitología Médica (IMPAM). Universidad de Buenos Aires- Consejo Nacional de Investigaciones Científicas y Técnicas (CONICET), Buenos Aires, Argentina; ^2^ Departamento de Bioquímica Clínica, Facultad de Farmacia y Bioquímica, Cátedra de Microbiología Clínica, Inmunología y Virología Clínica, Universidad de Buenos Aires, Buenos Aires, Argentina; ^3^ Instituto de Fisiopatología y Bioquímica Clínica (INFIBIOC), Universidad de Buenos Aires, Buenos Aires, Argentina; ^4^ División Cardiología, Hospital de Clínicas “José de San Martín”, Universidad de Buenos Aires, Buenos Aires, Argentina; ^5^ Departamento de Microbiología Parasitología e Inmunología, Facultad de Medicina, Universidad de Buenos Aires, Buenos Aires, Argentina; ^6^ División Infectología, Hospital de Clínicas “José de San Martín”, Universidad de Buenos Aires, Buenos Aires, Argentina

**Keywords:** interleukin 10, polymorphisms, Chagas disease, cardiomyopathy, meta-analysis, case-control study, association study

## Abstract

**Background:**

Chagas disease is a lifelong infection caused by the protozoa *Trypanosoma cruzi* endemic in Latin-America and emergent worldwide. Decades after primary infection, 20-30% of infected people develop chronic Chagas cardiomyopathy (CCC) while the others remain asymptomatic. CCC pathogenesis is complex but associated with sustained pro-inflammatory response leading to tissue damage. Hence, levels of IL-10 could have a determinant role in CCC etiology. Studies with Latin-American populations have addressed the association of genetic variants of IL-10 and the risk of developing CCC with inconsistent results. We carried out a case control study to explore the association between IL-10-1082G>A (rs18008969), -819C>T (rs1800871), -592A>C (rs1800872) polymorphisms and CCC in a population attending a hospital in Buenos Aires Argentina. Next, a systematic review of the literature and a meta-analysis were conducted combining present and previous studies to further study this association.

**Methods:**

Our case control study included 122 individuals with chronic *T. cruzi* infection including 64 patients with any degree of CCC and 58 asymptomatic individuals. Genotyping of IL-10 -1082G>A, -819C>T, -592A>C polymorphisms was performed by capillary sequencing of the region spanning the three polymorphic sites and univariate and multivariate statistical analysis was undertaken. Databases in English, Spanish and Portuguese language were searched for papers related to these polymorphisms and Chagas disease up to December 2021. A metanalysis of the selected literature and our study was performed based on the random effect model.

**Results:**

In our cohort, we found a significant association between TT genotype of -819 rs1800871 and AA genotype of -592 rs1800872 with CCC under the codominant (OR=5.00; 95%CI=1.12-23.87 P=0,04) and the recessive models (OR=5.37; 95%CI=1.12-25.68; P=0,03). Of the genotypes conformed by the three polymorphic positions, the homozygous genotype ATA was significantly associated with increased risk of CCC. The results of the meta-analysis of 754 cases and 385 controls showed that the TT genotype of -819C>T was associated with increased CCC risk according to the dominant model (OR=1.13; 95% CI=1.02–1.25; P=0,03).

**Conclusion:**

The genotype TT at -819 rs1800871 contributes to the genetic susceptibility to CCC making this polymorphism a suitable candidate to be included in a panel of predictive biomarkers of disease progression.

## Introduction

Chagas disease is one of the most widespread endemic diseases in Latin America being responsible for 9490 deaths (95% CI 5500–16 500) in 2019 (1). With an annual incidence of 28,000 cases in Central and South America, it is estimated that Chagas disease affects around six million people and causes nearly 12,000 deaths each year being around 65 million people are at risk of contracting the disease (2). Chronic Chagas disease is one of the impoverishment factors in rural areas of Latin America and responsible for 275 000 DALYs (184 000–459 000) in 2019 (1). In recent decades, a phenomenon of global dispersion of the disease has been observed, due to the migration of infected individuals from rural to urban areas within endemic countries and from endemic countries to non-endemic countries. Congenital transmission, blood transfusions, organ transplantation and laboratory accidents, transmission routes that do not require vector intermediation, are responsible for the global emergence of the disease.

Chagas disease is a condition with a wide spectrum of clinical outcomes. The majority of infected individuals remains asymptomatic for life (60-70%). Some, individuals will develop symptoms, predominantly cardiomyopathy but also digestive, or cardiodigestive symptoms. Progression to cardiomyopathy among infected individuals occurs at an annual rate of 1.85% to 7% (3). Chronic Chagas cardiomyopathy (CCC) pathogenesis is not completely understood but is believed to be multifactorial. Dysregulated inflammatory processes associated with parasite persistence result in progressive cardiac conduction anomalies, microvascular alterations leading to thrombosis, myocardial fibrosis, adverse left ventricle remodeling and heart failure (4–6).

Hence, one of the biggest challenges for physicians and researchers tackling this disease is to predict and prevent the establishment of the pathology in chronically infected individuals. It is therefore necessary to understand the factors that mediate clinical progression of susceptible individuals. In this line, host´s immune response appears to play a central role in the development of CCC and recent studies are helping to differentiate protective responses from pathogenic ones (7–11).

IL-10 is a cytokine with anti-inflammatory functions that regulates the immune response by limiting the production of other pro-inflammatory cytokines and, indirectly, the Th1 response by modulating the function of antigen-presenting cells (12, 13). This cytokine is produced by different cell types in response to infection by *T. cruzi* and other intracellular pathogens thus modulating immune mechanisms elicited against these pathogens (13–18). In late stages of Chagas disease, IL-10 participates in delaying the onset of CCC in infected individuals (19–21). A comprehensive analysis of the cytokine profile revealed that the decrease in IL-10 levels switches the immune response from the anti-inflammatory profile of asymptomatic patients to the pro-inflammatory one of cardiac patients (22–25).

Several studies have evaluated the association of human polymorphisms in genes encoding for cytokines with the progression of inflammatory, infectious, and autoimmune diseases as well as cancer. Such polymorphisms could be used as genetic biomarkers of susceptibility and severity of disease (26). Single nucleotide polymorphisms (SNPs) have been described upstream of the transcription start site of the IL10 gene (27). Three of them -1082 (rs1800896, transition from A to G), -819 (rs1800871, transversion from C to T and -592; rs1800872, transversion from C to T) are functional polymorphisms with an influence on the levels of cytokine production (28, 29). Low production genotypes are related to susceptibility to systemic lupus erythematosus and progression of HIV to AIDS among other conditions (30–33).

Association between variants of IL10 promoter region and the susceptibility to CCC has been found in a genetic study performed in a Brazilian cohort (34). However, studies in a Colombian cohort (35) and in Brazilian populations from other regions failed to confirm this association or showed only trends of association (36, 37). The heterogeneity of populations in Latin America as well as the small sample size are limitations for these association studies. Still, in view of the central role of IL-10 in Chagas disease progression we aimed to establish the association between IL-10 polymorphisms and the development of CCC in seropositive *T. cruzi* patients attending to a hospital in Buenos Aires, Argentina. Next, we performed a systematic review of the literature of studies on IL-10 polymorphisms and Chagas disease and conducted a meta-analysis to estimate a consensus association estimation.

## Materials and Methods

### Study Population

The project was submitted to and approved by the HCJSM ethics committee in accordance with the principles of the Declaration of Helsinki. All patients signed an informed consent after reading and understanding the study information prior to entering this study.

A cross-sectional study was carried out from January 2015 to January 2019. Patients attended by spontaneous demand to infectious diseases specialist and cardiologist at the Hospital de Clínicas José de San Martín of the University of Buenos Aires (HCJSM). Those patients who met the inclusion criteria and agreed to participate in the study were included. The inclusion criteria were patients over 18 years of age with a diagnosis of chronic infection by *T. cruzi* without risk of acute vectorial or transfusional infection or by intravenous drug abuse. Individuals with reactive anti *T. cruzi* antibodies by two of three distinct serological techniques were considered at chronic infection. The clinical-epidemiological data were included in a file completed by the physicians in charge. Patients with reactive serology for *T. cruzi* were classified according to the clinical form of the infection. Those without cardiac signs and symptoms by clinical evaluation and electrocardiogram without alterations were classified as asymptomatic (ASYM). Patients with associated cardiac signs and symptoms or presence of electrocardiographic abnormalities, altered echocardiography, cardiomegaly and/or placement of cardiac devices were included in the cardiomyopathy group (CCC). Patients with heart disease from non-Chagas disease ethology (congenital, hypertensive, ischemic and primary) diabetes mellitus, renal insufficiency, ​​immunocompromised patients or those with autoimmune or oncological diseases were excluded from the study.

Blood and serum samples used for molecular biology and immunological studies were labeled and identified by a group investigator. An alphanumeric code was used which later allowed the sample to be identified in the database. DNA was extracted from EDTA-anticoagulated whole blood (mixed 1: 1 with guanidine) using a commercial DNA Puriprep S-kit (Inbio Highway), following manufacturer’s protocol.

### IL-10 Genotyping

A PCR with the primers 5 ‘ATC CAA GAC AAC ACT ACTA A 3’ 5 ‘TAA ATA TCC TCA AAG TTC C 3’ was carried out to generate the amplification product (587 bp) that covered the 3 polymorphic sites (rs1800872, rs1800871 and rs1800896) of the IL-10 gene promoter region (28). PCR products were subjected to capillary sequencing and the complementarity with the sequence deposited in GenBank was confirmed (ACC N °: Z30175, ID: 3586).

### Statistical Analysis

Categorical and continuous data were expressed as percentages of total or as mean ± SD, respectively. Student’s t-test was used to determined differences in means while χ2 test was used for differences in categorical variables between groups. Logistic regression analysis was used to determine the odds ratios (OR) and 95% confidence intervals (95% CI) associated with CARD risk in four genetic models (codominant, dominant, recessive, overdominant) taking the major allele as the reference. Binary logistic regression was also used to adjust for confounders. Statistical significance was set at P < 0.05. Statistical analysis was performed on STATA 13 (StataCorp).

Genotypes of the three SNPs were tested for Hardy–Weinberg equilibrium (HWE) in the control and cases using the χ2 test. Pairwise linkage disequilibrium (LD) (D’ and r^2^) was estimated using Arlequin 3.11 and haplotype reconstruction was performed by the expectation maximization method (Haploview 4.2).

### Systematic Review and Meta-Analysis

#### Search Strategy, Study Selection and Data Extraction

The PRISMA 2020 guidelines were used to design a meta-analysis. A comprehensive search of the literature published up to December 2021 in English, Spanish or Portuguese was performed on the PubMed, LILACS and Scopus online databases. The searching terms used were as follows: (“Chagas” OR “Chagas disease” OR “Trypanosoma cruzi” OR “American trypanosomiasis”) AND (“polymorphism” OR “single nucleotide polymorphism” OR “SNP” OR “variant”) AND (“interleukin-10” OR “IL-10” OR “IL10”). Eligible studies were those case control-studies focused on the associations between IL-10 -1082G>A, -819C>T, -592A>C polymorphisms and risk of chronic Chagas cardiomyopathy in Chagas disease patients. Reference lists in identified articles and reviews were also searched manually to identify additional eligible studies. Additional inclusion criteria were 1) diagnosis of CCC established by a cardiologist with a minimum criterion of electrocardiogram and chest X-ray and 2) availability of either number or odds ratio of alleles genotypes or haplotypes. Exclusion criteria were prospective studies, case reports, meeting abstracts, repeated publication, or overlapped data.

The relevant data extracted from the eligible publications by 2 independent researchers (CAS and JQB) included: first authors name, publication date, country of origin, genotyping methods, sample size of cases and controls, allele

and genotype frequency of IL-10 -1082G>A, -819C>T, -592A>C polymorphisms in cases and controls, minor allele frequencies (MAF) and Hardy-Weinberg equilibrium (HWE) in healthy controls.

#### Quality Assessment and Risk of Bias

The quality of the studies included in the analysis was assessed applying the Newcastle Ottawa scale (38) independently by two assessors (CAS and JQB). Only high-quality studies were included in the meta-analysis (scores above 5 points on a 10-point Newcastle Ottawa scale).

#### Meta-Analysis

Effect sizes were calculated from raw data as unadjusted odds ratio (OR) with 95% confidence interval (CI) to assess the strength of the association of IL-10 -1082G>A, -819C>T or -592C>A polymorphisms and risk of CCC among individuals with chronic *T. cruzi* infection.

The association of IL-10 -1082G>A, -819C>T and -592C>A polymorphism was estimated under five genetic models, i.e., homozygote (MM vs. mm), heterozygote (Mm vs. mm), dominant (MM+Mm vs. mm), recessive (MM vs. Mm+mm) and the over-dominant (MM+mm vs Mm).

As we anticipated considerable between-study heterogeneity, we used the Mantel-Haenszel method to pool effect sized under the random-effects model. The heterogeneity variance τ2 was calculated with the Paule-Mandel estimator for binary effect size data (39). We used Knapp-Hartung adjustments (40) to calculate the confidence interval around the pooled effect.” HWE in control groups were assessed using the goodness-of-fit Chi-square test and a p-value <0.05 was considered as significant disequilibrium.

Statistical tests for the meta-analysis were performed in a R environment version 4.0.3 using the “meta” and “metafor” packages (41). All tests were two-sided, and the P< 0.05 was considered statistically significant.

## Results

### Characteristics of Participants

Our study population comprises fewer males than females. However, gender was equally distributed among cases and control individuals (**Table 1**). Mean age distribution was a significantly different between cases and controls (P<0,0001). Although more than 50% of patients in either group were > 50 year. The case to control ratio was 1,18 (cases=52,5%; controls =47,5%).

**Table 1:** Demographic Parameters of Chronic Chagas Cardiomyopathy (CCC) Patients and Control Group (ASYM).

	ASYM n = 58	CCC n = 64	P value (*X* ^2^)
**Gender**			0.199 ^a^
Female	46 (79.3%)	38 (65.5%)	
Male	12 (20.7%	26 (34.5%)	
**Age**			
Range (Min-Max)	57 (19-76)	60 (26-86)	
Mean +-SD	46,31 +- 14,15	61.17 +- 11.00	<0,0001^b^
Argentine	31 (53,4%)	52 (81,3%)	0,0017^b^

a Fisher’s exact test.

b Unpaired t test with Welch’s correction.

### Association Studies

All participants were genotyped for the three SNPs (rs1800896; rs1800871; rs1800872) being genotype frequencies of these SNPs in controls in Hardy-Weinberg equilibrium (P=0.70; P=0.36; and P=0.36 respectively). Minor allele frequencies calculated for each SNP were above 20% and higher in the cases than in control individuals for the three SNPs. Allele and genotype distributions of these polymorphism are summarized in **Table 2**. The association of these SNPs with increased risk of CCC was evaluated under four genetic models: Codominant; dominant, recessive model and over dominant model. Crude odds ratios revealed a significant association between TT genotype of -819 rs1800871 and AA genotype of -592 rs1800872 with CCC under the codominant and the recessive models. Adjusting for gender did not modify the OR but adjusting for age increased it.

**Table 2:** Association of SNPs with CCC in cases and controls.

SNP	Model	Genotype	ASYM n (%)	CCC n (%)	OR (CI95%)	P	Age adjusted OR (CI95%)	P
-1082 rs1800896	Codominant	AA	28 (50)	36 (54,5)	1	0,71		
		AG	24 (42,9)	24 (36,4)	0,77 (0,37-1,62)	0,56		
		GG	4 (7,1)	6 (9,1)	1,16 (0,32-3,93)	>0,99		
	Dominant	AA	28 (50)	36 (54,5)	1			
		GG+GA	28 (50)	30 (45,5)	0,83 (0,39-1,72)	0,71		
	Recessive	GA+AA	52 (92,9)	60 (90,9)	1			
		GG	4 (7,1)	6 (9,1)	1,3 (0,35-4,25)	0,75		
	Overdominant	GG+AA	32 (57,1)	42 (63,6)	1			
		GA	24 (42,9)	24 (36,4)	0,76 (0,37-1,55)	0,57		
-819 rs1800871	Codominant	CC	31 (55,4)	33 (50)	1			
		TC	23 (41,6)	23 (34,8)	0,93 (0,44-1,99)	>0,99		
		TT	2 (3,6)	10 (15,2)	**5,00 (1,12-23,87)**	**0,04**	**5,76 (1,06-31,13)**	**0,04**
	Dominant	CC	31 (55,3)	33 (50)	1			
		TT+TC	25 (41,1)	33 (50)	1,24 (0,59-2,60)	0,58		
	Recessive	TC+CC	54 (96,4)	56 (84,8)	1			
		TT	2 (3,6)	10 (15,2)	**5,37 (1,12-25,68)**	**0,03**	**6,45 (1,31-31,59)**	**0,02**
	Overdominant	TT+CC	33 (55,4)	43 (65,2)	1			
		TC	23 (41,6)	23 (34,8)	0,76 (0,37-1,55)	0,57		
-592 rs1800872	Codominant	CC	31 (55,4)	33 (50)	1			
		AC	23 (41,6)	23 (34,8)	0,93 (0,44-1,99)	>0,99		
		AA	2 (3,6)	10 (15,2)	**5,00 (1,12-23,87)**	**0,04**	**5,76 (1,06-31,13)**	**0,04**
	Dominant	CC	31 (55,3)	33 (50)	1			
		AA + AC	25 (41,1)	33 (50)	1,24 (0,59-2,60)	0,58		
	Recessive	AC +CC	54 (96,4)	56 (84,8)	1			
		AA	2 (3,6)	10 (15,2)	**5,37 (1,12-25,68**	**0,03**	**6,45 (1.31-31,59)**	**0.02**
	Overdominant	AA+CC	33 (55,4)	43 (65,2)	1			
		AC	23 (41,6)	23 (34,8)	0,76 (0,37-1,55)	0,57		

The bold text represents the statistically significant results (p<0.05).

**Figure 1:**
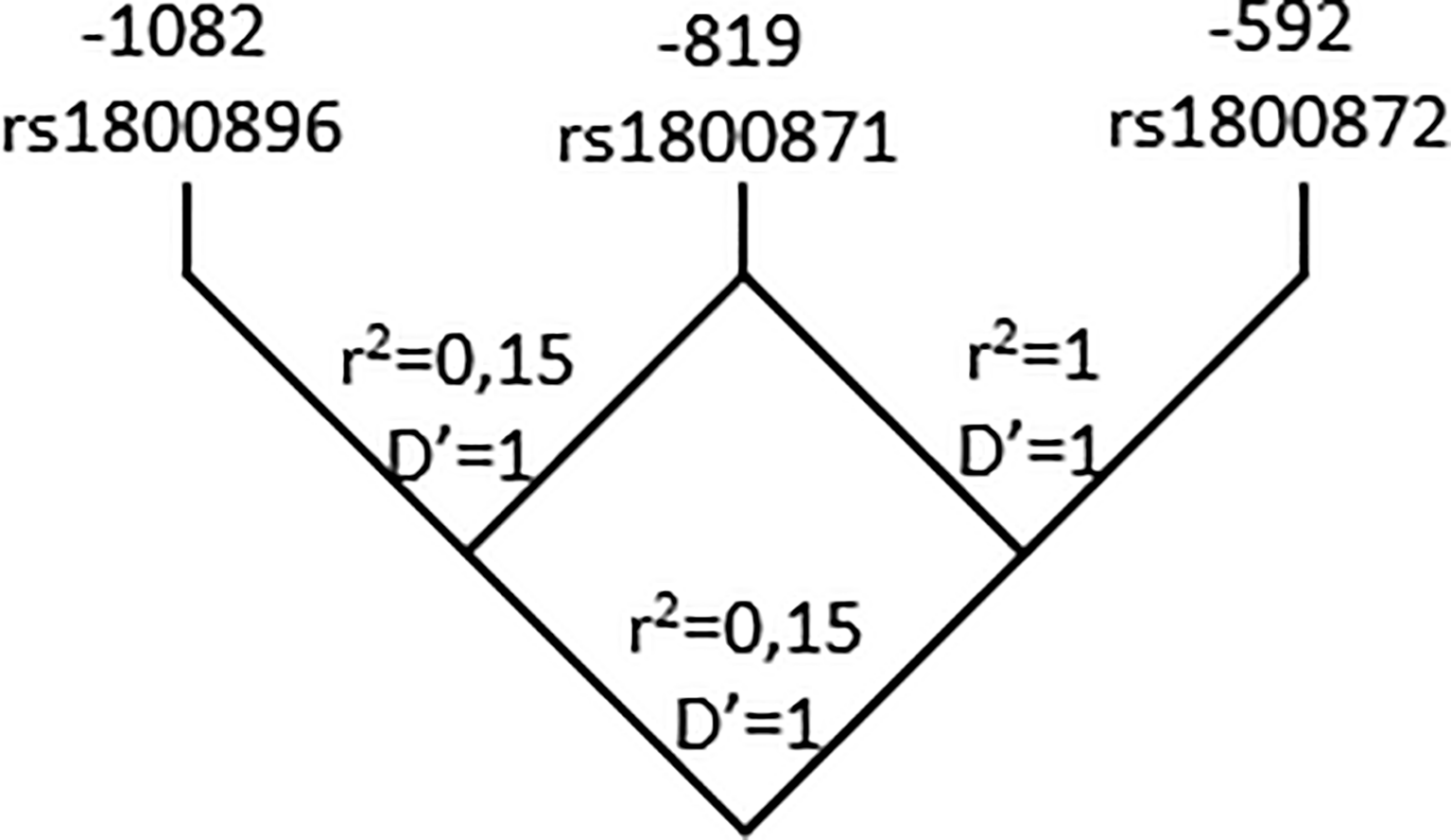
Pairwise LD map for SNPs rs1800896; rs1800871; rs1800872 across *IL-10* region. Lewontin’s D’ measure and r^2^ values of LD are shown.

As reported in previous studies with subjects from diverse demographic characteristics, the three SNPs are in LD. The D and r^2^ value between -1082 rs1800896 and - 819 rs1800871 are 1 and 0.15 respectively. Yet, these SNPs cannot substitute each other. Regarding - 819 rs1800871 and -592 rs1800872 they are in complete LD (r^2 =^ 1; D’=1) thus retrieving similar genetic results (**Figure 1**). The three SNPs constructed three haplotypes (“GCC”, “ACC”, “ATA”) in the promoter region of the IL-10 gene that combined retrieved six genotypes. The homozygous genotype ATA was significantly associated with increased risk of CCC under a crude logistic-regression model (**Table 3**). Adjusting for gender did not modify the OR but adjusting for age further increased the association.

**Table 3:** Association of the genotypes with CCC in cases and controls.

Genotype	ASYM n (%)	CCC n (%)	OR (CI95%)	P	Age adjusted OR (CI95%)	P
AYM	14 (25,0)	13 (19,7)	0,73 (0.29 - 1.77)	0,71		
RYM	9 (16,1)	10 (15,2)	0,93 (0.36 - 2.36)	>0.99		
RCC	15 (26,8)	14 (21,2)	0,73 (0.31 - 1.68)	0,52		
GCC	4 (7,1)	6 (9,1)	1,30 (0.35 - 4.25)	0,75		
**ATA**	**2 (3,6)**	**10 (15,2)**	**5,37 (1,12- 25,68)**	**0,03**	**6,45 (1.31-31,59)**	**0,02**
ACC	12(21,4)	13 (19,7)	0,89 (0.38 - 2.07)	0,82		

### Meta-Analysis

#### Description of Studies

The searching strategy used for the online databases identified 23 records at Pubmed, 29 records at Scopus and 14 records at Lilacs. In agreement with the eligibility criteria, the relevant articles were checked according to the inclusion and exclusion criteria thus retrieving 4 case control studies focusing on the association of IL-10 polymorphisms and CCC to be included in the meta-analysis. Of these studies, and our present study, 5 studies involving 851 cases and 443 controls were on -1082G>A, 4 studies involving 754 cases and 385 controls were on -819C>T and two studies involving 196 cases and 186 controls were on -592C>A polymorphism. The distribution of genotypes in controls was consistent with Hardy-Weinberg equilibrium (P>.05) in all studies. The quality of the included studies was adequate being the mean Newcastle-Ottawa score 6.4 (**Table 4**).

**Table 4:** Methodological quality of studies included in the meta-analysis, based on the Newcastle - Ottawa quality assessment scale for case control studies.

	Study	Present study	Costa	Flores	Alvarado-Arnez	Frade-Barros
**Selection**	Adequate definition of cases	1	1	1	1	1
	Representativeness of the cases	1	1	1	1	1
	Selection of controls	1	0	1	0	1
	Definition of controls	1	1	1	1	1
**Comparability**	Control for important factor or additional factor	1	1	0	1	1
**Exposure**	Ascertainment of exposure	0	0	0	0	0
	Same method of ascertainment for cases and controls	1	1	1	1	1
	Non-Response rate	1	1	1	1	1
	**Total score**	**7**	**6**	**6**	**6**	**7**

#### Quantitative Data Synthesis

**Figure 2:**
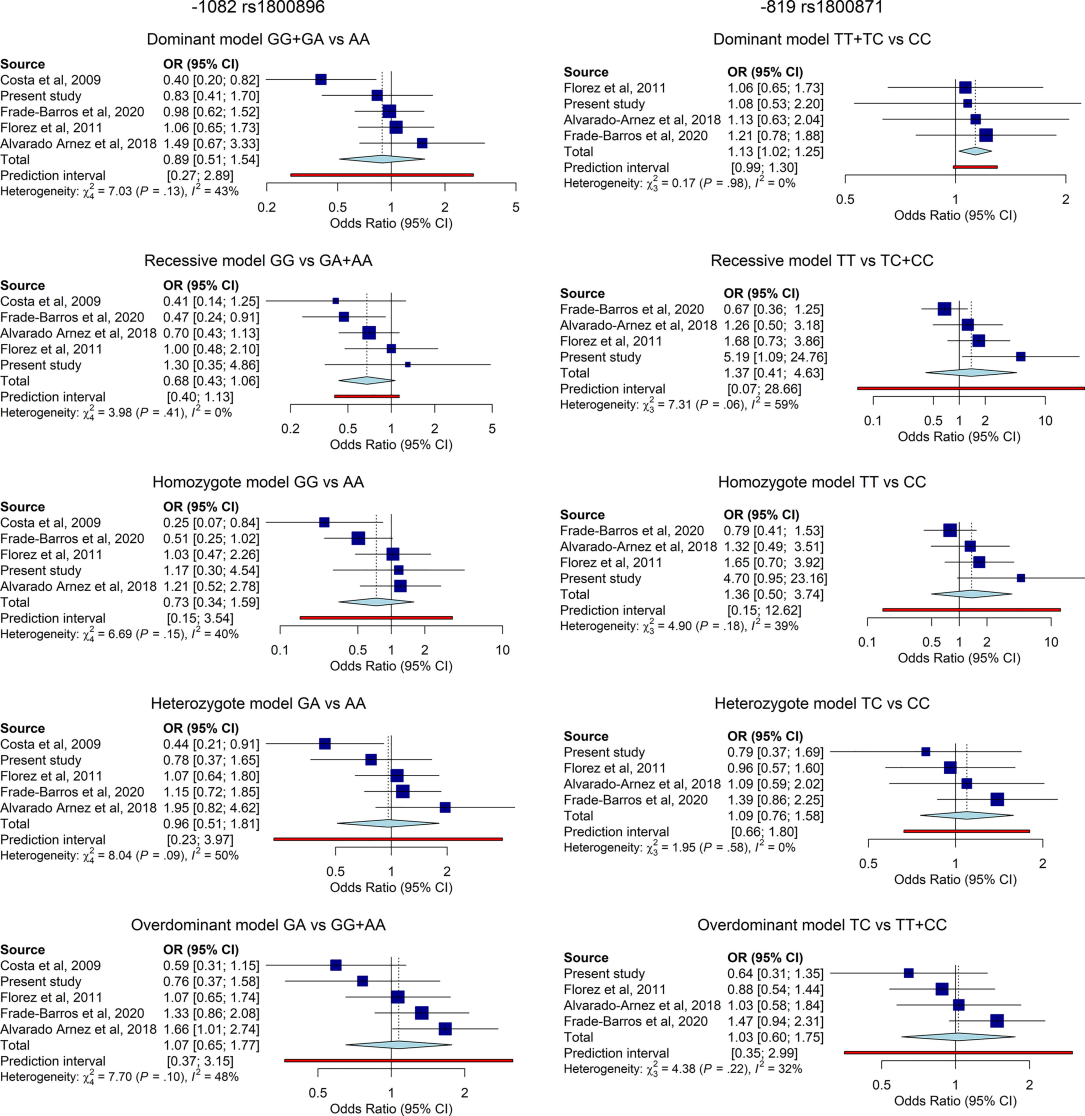
Forest plot and pooled OR for the association of IL-10 -1082G>A (left), and (right) polymorphisms and risk of CCC among individuals with chronic *T. cruzi* infection under five different genetic models. Horizontal lines represent the 95% confidence intervals around the point estimates for each study and the blue squares represent the OR whose size are proportional to the weight given to each study. The light blue diamonds represent the pooled effect and its 95% confidence intervals for all studies according to the random-effects model. Adjustment for age or gender was not performed due to the lack of information on such covariates.

The overall results are summarized in the forest plots (**Figure 2**). Heterogeneity between studies was moderate to low. The significance of the association was tested under five different genetic models. For each genetic model, the OR and CI95% and the weight given to each study are shown (blue squares and horizontal lines) and the pooled effect sized under the random-effects model and CI95% (light blue diamonds). Regarding 1082G>A rs1800896 (lef panel), the analysis comprised 5 studies involving in total 851 cases and 443 controls. The meta-analysis did not show significant association for IL-10 -1082G>A and CCC risk among *T. cruzi* infected individuals. Still, a tendency towards protection of the GG genotype was noticeable under dominant, recessive and homozygote models (left panel). Regarding -819C>T rs1800871 (right panel), the analysis comprised 4 studies involving in total 754 cases and 385 controls. The overall results of the meta-analysis indicated that the TT genotype of -819C>T could be associated with increased CCC risk according to the dominant model (OR=1.13, 95% CI=1.02–1.25, P=0,03, **Figure 2**).

## Discussion

Genetic variations in the IL-10 gene promoter influence the levels of IL-10 production and are associated with the outcome of infectious diseases and inflammatory disorder. The case control study presented here shows an association in our population between the -819C>T (rs1800871) polymorphism in IL-10 and an enhanced risk of developing CCC in individuals with chronic *T. cruzi* carrying the TT genotype under the codominant and recessive genetic models. Frade-Barros and coworkers (36) also found a significant association for this marker -819 (rs1800871) and severe CCC when compared to ASYM under the overdominant model but not when comparing between the ASYM subjects and the whole CCC group in a Brazilian cohort. Studies evaluating this same marker in cohorts from Colombia (35) and other regions of Brazil (34, 37), did not found such association. Regarding -1082 rs1800896 marker, Costa (34) and Alvarado-Arnez (37) found for this marker an association between AA genotype and AA and AG genotypes (dominant model) respectively and CCC. In our cohort, in line with other studies from Colombia and Brazil, we observed no association of this marker and risk of progression to CCC.

LD analysis of our study population is in accordance with results from ensembl.org which show a strong LD (r^2 =^ 0,208745) between -1082 and -819 or -592 and a complete LD (r^2 =^ 1) between -819 and -592. As observed by diverse reports in Caucasian and Asian populations, only three out of eight possible haplotypes (GCC, ACC and ATA) segregate in our population. Previous work identified the same haplotypes in a Colombian population but found no association with CCC (35). Here, the combination of these SNPs revealed that the homozygous genotype ATA is associated with CCC. Being these SNPs in LD, this result is in accordance with the association found between the TT genotype at -819C>T (rs1800871) and CCC. Of note, work from Costa (34) which shows an association between AA carriers in -1082 G>A (rs1800896) did not evaluate the -819C>T position. It is tempting to postulate that, in the Brazilian population, the strength of the association is due to the strong LD in their population between -819C>T (rs1800871) and -1082 G>A (rs1800896) as found by Frade-Barros and coworkers (36). We found the same enhanced risk of developing CCC in individuals carrying the AA genotype of -592 rs1800872 in the codominant and recessive genetic models as in those carrying the TT variant at-819 (rs1800871). This finding was expected as both markers are in complete LD.

Some reports have suggested sex-related differences in the outcome of Chagas disease being male gender associated with worse prognosis (42, 43). Here, stratification by gender does not modify the strength of the association thus suggesting that this variable does not influence the increased risk of CCC caused by genetic variants of IL-10 promoter. Conversely, adjusting by age further increased the risk of CCC by some variants of IL-10 promoter. The confounding effect of age on this association is in line with the natural history of Chagas disease as the incidence of cardiopathies increases as infected individuals age (44). This finding confirms a generalized limitation of associations studies related to CCC: Sample size should be adequate to undertake subgroup analysis or the study subjects should be above 50 years of age were the average impact of Chagas disease on target organs is noticeable in order to avoid including cases in the control group.

The genetic polymorphisms regulate both constitutive and induced levels of IL-10 (28). GCC haplotype is associated with high IL-10 production at transcriptional and protein level in the basal state and upon lymphocyte stimulation whereas the ATA haplotype is detected in low IL-10 secretors (29, 45, 46). In our study, the homozygous genotype ATA is associated with increased risk of CCC. Studies in various contexts proposed a protective role of high-levels of IL-10 against Chagas disease progression, as these levels can attenuate the inflammatory tissue damage elicited by persistent infection with the protozoa (19–24). The association between low levels of IL-10 production and progression Chagas disease cardiomyopathy described in this study provides a rationale for immunomodulatory treatments for chronic Chagas disease potentiating IL-10 driven immunomodulatory functions (11).

Disagreement between the case control studies performed so far could be due to the small size of studies and the possible impact of genetic background of different populations. Therefore, we performed this meta-analysis of the role of IL-10 -1082G>A, -819C>T and -592C>A polymorphisms in the susceptibility to CCC in chronically infected individuals. Considering the complete linkage disequilibrium between -819C>T and -592C>A polymorphisms we limited our study to IL-10 -1082G>A and -819C>T polymorphisms.

Pooling data from our current study and previous studies from different Latin American populations revealed a between-study heterogeneity which ranged from low to moderate according to the genetic model and the studied SNP. Hence, according to the random-effect model, the TT genotype of -819C>T was associated with the risk of CCC risk under the dominant model validating case control. Regarding the -1082G>A, the meta-analysis did not show nominally statistical differences but revealed a tendency towards an increased risk of CCC for the A allele in line with the work from Costa (34). The strong LD between the three polymorphic positions make us suggest that -819C>T could be the most informative SNP for future association studies.

We are aware of the limitations from our meta-analysis as confounding factors which correlate with CCC susceptibility, such as age, sex, endemic region, were not taken into consideration. Moreover, even though the pathogenesis of CCC is complex and multifactorial the associations between IL-10 polymorphisms and CCC susceptibility were analyzed independently without contemplating neither other host factors as the production of other immunoregulatory cytokines (i.e. IL-4; TGF-beta or IL-35) nor gene-environment, gene-gene interactions, and the parasite genetics. Still, this first meta- analysis may improve our understanding of the role of IL-10 -1082G>A, -819C>T and -592C>A polymorphisms in susceptibility to CCC.

In summary, TT at -819 contributes to the genetic susceptibility to CCC making this polymorphism a suitable candidate to be included in a panel of predictive biomarkers of disease progression. A most needed tool to improve the follow up and clinical management of individuals chronically infected with *Trypanosoma cruzi*.

## Data Availability Statement

The original contributions presented in the study are included in the article/supplementary material. Further inquiries can be directed to the corresponding author.

## Ethics Statement

The studies involving human participants were reviewed and approved by Comité de ética del Hospital de Clínicas José de San Martin. The patients/participants provided their written informed consent to participate in this study.

## Author Contributions

CAS and SR contributed to conception and study design. CAS gave instructions, supervision and wrote the original draft. CAS, SR, and JQB performed the statistical analysis and interpreted data. AG and JQB contributed to the methodology and data curation and edited the manuscript. AG, SR, LGV, AT, DS, PR, MGR, and RNA collected data and edited the manuscript. AG, LGV, RNA, JQB, and AT organized the database. All authors contributed to the article and approved the submitted version.

## Funding

This study was supported by Secretaría de Ciencia y Técnica, Universidad de Buenos Aires (20020170100289BA to CAS and 20720190200012BA to LGV), Fondo Nacional de Ciencia y Tecnología (PICT-2014 0733 to CAS) and Consejo Nacional de Investigaciones Científicas y Técnicas (PIP 2015-0733 to CAS). The funder was not involved in the design of the study and collection, analysis and interpretation of data or in writing the manuscript.

## Conflict of Interest

The authors declare that the research was conducted in the absence of any commercial or financial relationships that could be construed as a potential conflict of interest.

## Publisher’s Note

All claims expressed in this article are solely those of the authors and do not necessarily represent those of their affiliated organizations, or those of the publisher, the editors and the reviewers. Any product that may be evaluated in this article, or claim that may be made by its manufacturer, is not guaranteed or endorsed by the publisher.
